# The role of mitoxantrone hydrochloride in lymph node harvesting and parathyroid gland identification in conventional and endoscopic thyroidectomy

**DOI:** 10.7150/ijms.126168

**Published:** 2026-03-25

**Authors:** Yifan Liu, Di Zhou, Rong Cong, Botao Sun, Fada Xia, Xinying Li

**Affiliations:** 1 Department of General Surgery, Xiangya Hospital, Central South University, No. 87 Xiangya Road, Changsha, 410008, China.; 2 National Clinical Research Center for Geriatric Disorders, Xiangya Hospital of Central South University, Changsha, Hunan, China.

**Keywords:** mitoxantrone hydrochloride injection, endoscopic thyroidectomy, central neck dissection, parathyroid glands

## Abstract

**Introduction:**

This retrospective study investigates the role of Mitoxantrone Hydrochloride Injection (MHI) in enhancing lymph node yield and protecting the parathyroid glands (PGs) in conventional and endoscopic thyroid surgery.

**Methods:**

A retrospective analysis was performed on 397 thyroid cancer patients who underwent surgery from May 2023 to May 2024. The patients were divided into MHI and control groups based on the intraoperative use of MHI. Clinical data, including lymph node harvest and PGs identification, as well as perioperative parathyroid hormone and calcium levels, were reviewed.

**Results:**

The study included 258 patients in the conventional surgery cohort and 139 in the endoscopic cohort (transoral, axillary, and periareolar approaches). No MHI-related complications were observed. In the conventional surgery cohort, MHI increased the lymph node yield per unilateral central neck dissection from 4.59 to 5.89, and in the endoscopic cohort, from 4.15 to 5.29. No difference was observed in the identification of superior PGs between groups. MHI enhanced the identification of inferior PGs from 42.45% to 60.87% in the conventional surgery cohort and from 41.00% to 60.92% in the endoscopic cohort. However, during the follow-up period, there was no significant difference in the incidence of hypoparathyroidism between the groups.

**Conclusions:**

MHI is a safe and effective adjunct for thyroid surgery, improving lymph node detection and inferior PGs exposure, although its measurable impact on postoperative parathyroid function in a high-volume center appears limited.

## Introduction

The incidence of thyroid cancer has been rising significantly, with most newly identified cases being papillary thyroid cancer (PTC) 1. Surgery confers a curative effect in the majority of patients with well-differentiated thyroid cancer 2. The anterior cervical approach has long been regarded as the gold standard for thyroid surgery 3. Minimally invasive approaches, such as transaxillary, transoral, transareola, bilateral axillo-breast, and subclavian techniques, have gained recognition for their safety and cosmetic advantages 4,5. PTC characteristically metastasizes to locoregional lymph nodes, and postoperative recurrence is clinically prevalent 6,7. Lymph node yield (LNY) and metastatic lymph node count in surgical specimens are critical prognostic indicators for predicting the risk of disease recurrence 8-10. Therefore, accurate lymph node dissection is essential for comprehensive management of patients. Hypoparathyroidism is a common complication following thyroidectomy, and its risk is significantly influenced by the extent of thyroidectomy, surgeon expertise, and intraoperative surgical techniques 11.

Currently, various contrast dyes are widely used to facilitate lymph node tracing and parathyroid glands (PGs) identification, including carbon nanoparticle (CN), indocyanine green (ICG), methylene blue (MB), and others. Mitoxantrone Hydrochloride Injection (MHI) is a novel lymph node tracer with high specificity for the lymphatic system. In thyroid surgery, local injection of MHI stains lymph nodes in the drainage area, enabling effective lymph node tracing while preserving negative contrast for the PGs 12.

Only a few studies have reported the use of MHI in thyroid cancer. It remains unclear whether MHI can improve lymph node visualization during standard central neck dissection (CND) in both conventional and endoscopic thyroid surgery. Given the variable anatomical locations of the superior and inferior PGs, the differential effects of MHI on the exposure and identification of these two PG subsets remain to be elucidated. This study aims to clarify the role of MHI in enhancing lymph node visibility in thyroid surgery, particularly in endoscopic procedures, and will conduct further analysis of the sizes and metastatic numbers of the identified lymph nodes. Additionally, the impact of MHI on the incidence of postoperative hypoparathyroidism will be evaluated, with a specific focus on its role in facilitating the exposure of the superior and inferior PGs during surgery.

## Methods

### Patients and data collection

Thyroid cancer patients who underwent surgery at Xiangya Hospital of Central South University between May 2023 and May 2024 were retrospectively enrolled. Inclusion criteria: a. Thyroid nodes were diagnosed as PTC by postoperative paraffin pathology; b. Patients underwent thyroidectomy and CND; c. Patients were aged between 18 and 70 years. Exclusion criteria: a. Previous history of thyroid surgery; b. Patients with preoperative hypoparathyroidism or hyperparathyroidism; c. Patients with a history of serious drug allergy or allergy to the study drug; d. Patients with serious underlying diseases. MHI implementation was initiated in our hospital in November 2023, with intraoperative MHI administered to all thyroid surgery patients thereafter. Eligible patients meeting the inclusion and exclusion criteria in the 6 months pre- and post-implementation were enrolled in this study. According to MHI use, patients were divided into MHI and control groups. To clarify potential differences in the utility of MHI, data from patients who underwent conventional open thyroid surgery and those who underwent endoscopic thyroid surgery were analyzed separately. A total of 258 patients underwent conventional thyroid surgery, of whom 120 were in the MHI group and 138 in the control group. Similarly, 139 patients underwent endoscopic thyroid surgery, with 65 in the MHI group and 74 in the control group.

We retrospectively collected general characteristics, surgical-related and postoperative follow-up data, which included general surgical-related information such as the surgical approach, extent of surgery, operative duration, intraoperative PG visualization, the count of lymph nodes identified postoperatively; short-term postoperative complications; and postoperative monitoring data comprising measurements of parathyroid hormone (PTH) concentrations and serum calcium levels. Hypoparathyroidism is defined as a PTH level below 15 pg/mL, regardless of the presence or absence of clinical symptoms associated with hypocalcemia. Transient hypoparathyroidism refers to the recovery of serum PTH levels to the normal range (> 15 pg/mL) within 6 months after surgery; permanent hypoparathyroidism is defined if serum PTH levels remain persistently below the normal value beyond 6 months postoperatively. All enrolled patients were followed up for more than 6 months, with a median follow-up duration of 9 months. This study was approved by the Medical Ethics Committee of Xiangya Hospital, Central South University (approval number: 2024101319). Written informed consent was obtained from the enrolled patients.

### Mitoxantrone hydrochloride injection for tracing

Mitoxantrone, a chemotherapeutic drug, is primarily used to treat hematological malignancies. Notably, mitoxantrone is dark blue and exhibits lymphatic system tropism. Nanoparticle delivery technology has been developed into a novel lymphatic tracer known as Mitoxantrone Hydrochloride Injection for Tracing. The capillary endothelial space of the human body is 30-50mm, and the capillary lymphatic endothelial space is 120-500nm. Following local injection, MHI self-assembles into 100-nm nanocrystals under physiological conditions. This allows the nanocrystals to selectively enter the capillary lymphatic vessels through endothelial spaces and macrophages. They are subsequently transported via lymphatic drainage and accumulate in regional lymph nodes, where they remain for an extended period. This process stains the lymph nodes blue, enabling effective lymphatic tracing 13. In this study, MHI for Tracing was provided by Shenzhen China Resources Jiuchuang Pharmaceutical Co., Ltd.

### Surgical procedure and MHI injection

**Figure 1:**
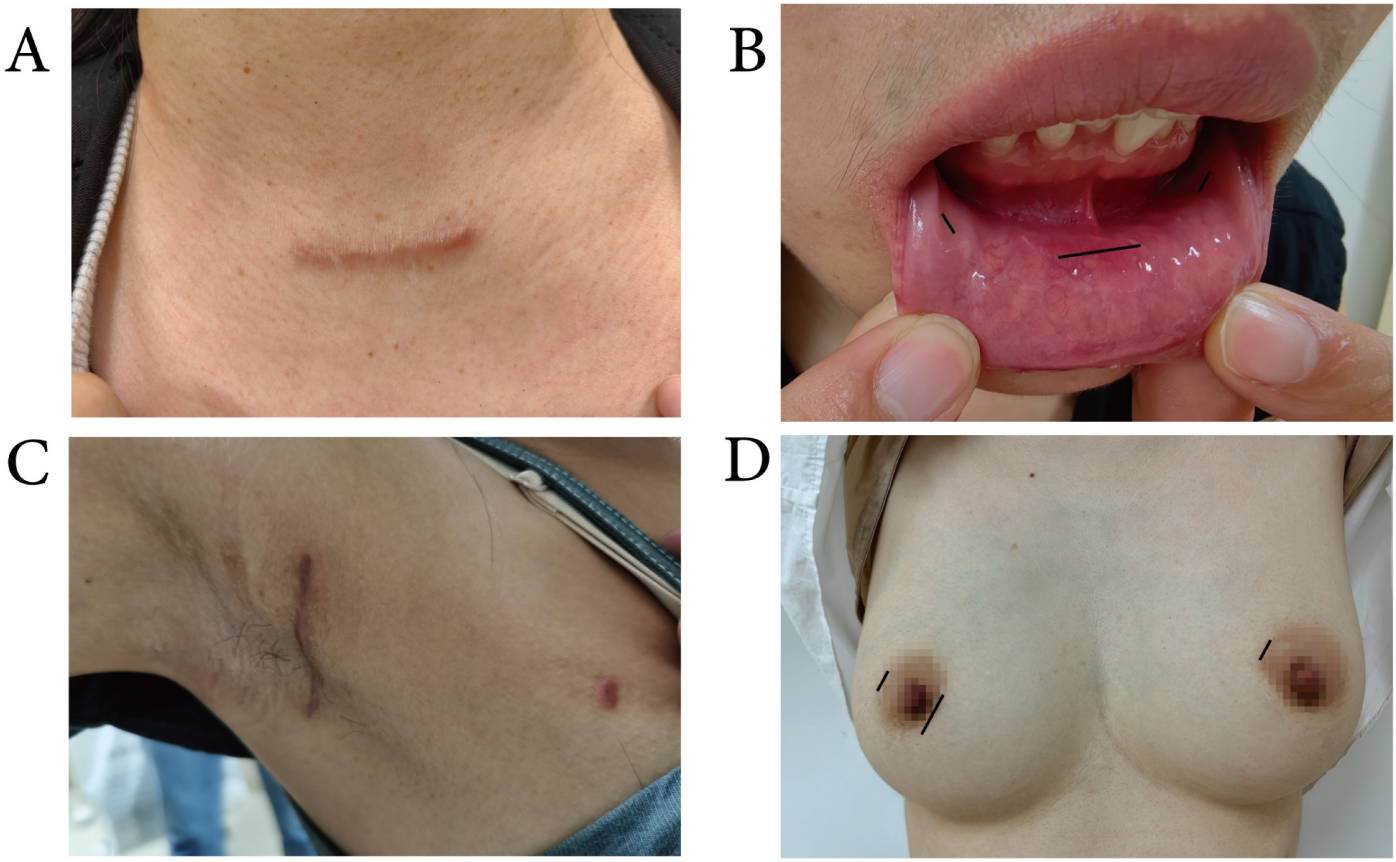
** Surgical incisions. A:** A 5 to 6 cm Kocher incision for conventional surgery. **B:** One 10-mm incision at the midline and two 5-mm incisions between the canine and the first premolar tooth for TOETVA. **C:** A 4-cm length incision behind the anterior axillary fold and a 5-mm incision on the vertical line of the TAT. **D:** One 10-mm incision at the right areola and two 5-mm incisions at the bilateral areola for ETBAA.

For conventional surgery, a 5 to 6 cm Kocher incision is made 1- 2 cm above the suprasternal notch, followed by lobectomy or total thyroidectomy with CND performed according to standard procedures. Endoscopic surgeries included transoral endoscopic thyroidectomy via the vestibular approach (TOETVA), transaxillary approach thyroidectomy (TAT), and endoscopic thyroidectomy via bilateral areola approach (ETBAA) (Figure 1).

TOETVA: This procedure followed a technique similar to that described in a previous study 14. Surgical incisions were made in the oral vestibule. A 10 mm incision is made in the middle as a viewing hole, and two 5 mm incisions are made between the canines and the first premolars as operating holes. The surgical pathway was established, extending down to the sternal notch, with its lateral boundary defined by the sternocleidomastoid muscles. CO_2_ gas was used to maintain working space at a pressure of 6- 8 mmHg*.* Thyroidectomy and CND were performed in a manner similar to that of conventional surgery.

TAT: The surgical steps were previously described in an earlier study 15. In brief, a 4-cm incision was made just posterior to the anterior axillary fold for the laparoscope and one surgical instrument, with an additional 5-mm accessory incision made vertically aligned with the axillary incision for another instrument. Workspace was built using a standard three-step method. First, the pathway to the anterior neck was dissected. Second, the sternocleidomastoid muscle was longitudinally incised, and the sternal head of the sternocleidomastoid muscle was elevated using a retractor. Finally, the strap muscles were dissected laterally, with the retractor positioned beneath them to expose the thyroid lobe. Thyroidectomy and CND were performed in the following sequence: ligation of the surrounding blood vessels, ligation of the superior thyroid artery, identification of the recurrent laryngeal nerve, CND, and transection of the thyroid isthmus.

ETBAA: This procedure was described in a previous study 16. Three incisions were required: a 10-mm incision at the right areola to accommodate the laparoscope, accompanied by two 5-mm incisions at the bilateral areolae for the insertion of the operating instruments. CO_2_ gas was used to maintain the working space at a 6- 8 mmHg pressure. The isthmus of the thyroid gland, the trachea, and the common carotid artery were exposed sequentially. The thyroid gland was then removed, and CND was performed using the same techniques as in other surgeries.

Central neck dissection was performed in accordance with the 2022 Chinese Guidelines for the Diagnosis and Treatment of Thyroid Carcinoma 17. The dissection scope was defined as follows: the inferior boundary at the level of the upper edge of the innominate artery, the superior boundary at the level of the hyoid bone, and the lateral boundary at the medial edge of the common carotid artery, including the pretracheal region. For patients with cN1 stage disease, therapeutic bilateral CND was routinely performed. In contrast, for patients with cN0/cNx stage disease, prophylactic CND was conducted on the affected side.

**Figure 2:**
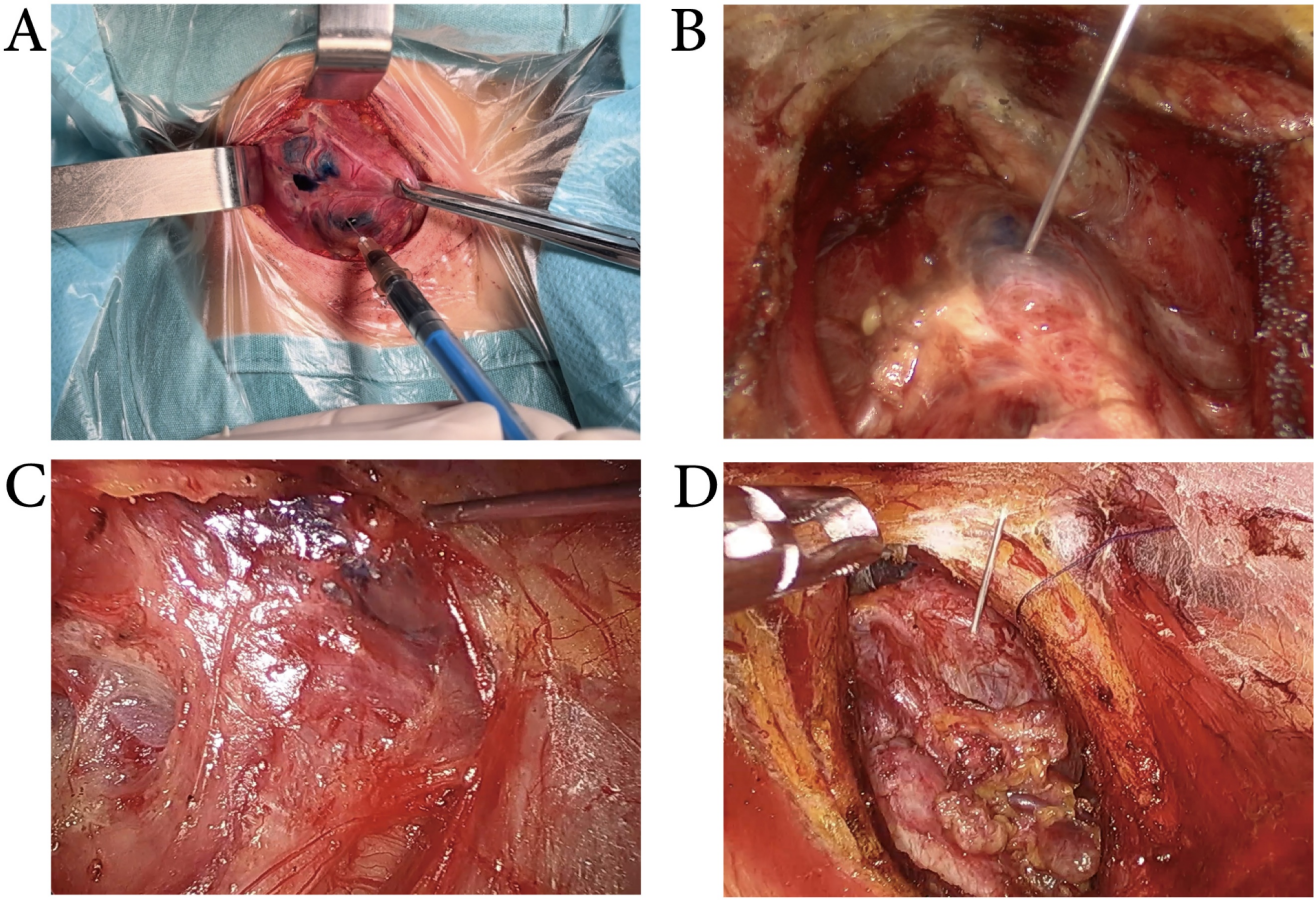
** Intraoperative MHI injection. A:** MHI was injected by using a 1-ml syringe in conventional thyroid surgery. **B, C&D:** MHI was injected by using a 1-ml syringe with an extended needle in transoral approach (B), transxillary approach (C), and bilateral areola approach (D) thyroid surgery.

Use of MHI: After exposing the thyroid anterior capsule, MHI (0.05- 0.1ml) was injected into the upper, middle, and lower parts of the normal thyroid tissue (the lobe with the tumor), with an injection depth of 2- 3mm. Following a 5-minute interval to allow MHI to infiltrate the draining lymph nodes, thyroidectomy and CND were performed. In conventional surgery, we injected MHI with a conventional 1-ml syringe. And in endoscopic surgery, we injected MHI with a 1-ml syringe with an extended needle (Figure 2).

### Statistical analysis

Continuous variables were expressed as means± standard deviations and analyzed using Student's t-test. Categorical variables were presented as frequencies and percentages (n, %) and analyzed using the chi-square test or Fisher's exact test. Statistical analyses were conducted using SPSS 27.0, with a significance threshold set at* p* < 0.05.

## Results

### Patient characteristics

This study included a total of 397 thyroid cancer patients, of whom 258 underwent conventional surgery (120 in the MHI group and 138 in the control group) and 139 underwent endoscopic surgery (65 in the MHI group and 74 in the control group). All procedures were performed successfully. The demographic date is summarized in Table 1. Baseline characteristics of patients who underwent conventional and endoscopic surgery were analyzed separately. Among the conventional surgery patients, no significant differences were observed between the MHI and control groups with respect to age, sex, tumor size, or tumor number. Similarly, in the endoscopic surgery group, the general characteristics were comparable between the two groups. In the conventional surgery cohort, 106 cases underwent unilateral surgery (49 in the MHI group and 57 in the control group), while 152 cases underwent total thyroidectomy (71 in the MHI group and 81 in the control group). In the endoscopic surgery cohort, 85 cases underwent unilateral surgery (37 in the MHI group and 48 in the control group), and 54 cases underwent total thyroidectomy (28 in the MHI group and 26 in the control group). In the endoscopic surgery cohort, the MHI group included 32 cases of transoral surgery, 22 cases of axillary approach surgery, and 11 cases of total mammary areolas approach surgery. The control group included 39 cases of TOETVA, 24 cases of TAT, and 11 cases of ETBAA. No significant differences were observed in operative time or surgical complications between the MHI and control groups. Postoperative hematoma occurred in one patient from the MHI group and two patients from the control group. Permanent hoarseness caused by tumor invasion of the recurrent laryngeal nerve was observed in two patients from each group, all of whom had undergone conventional thyroidectomy.

### MHI enhances the lymph node yield with a diameter < 5mm

**Figure 3:**
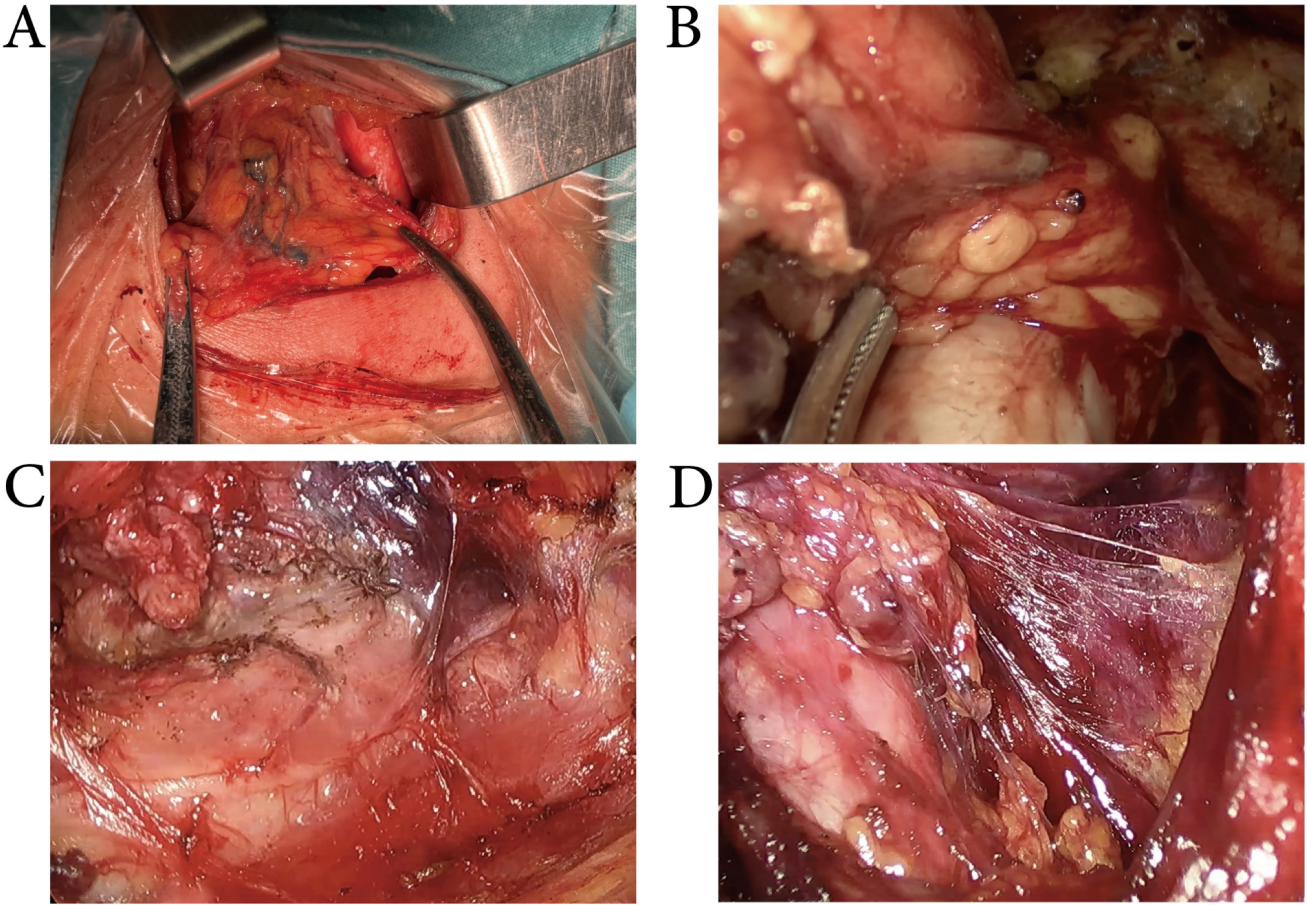
** The use of MHI during CND.** The lymph nodes were stained blue. MHI exhibited good staining effects in conventional (A), transoral approach (B), transxillary approach (C), and bilateral areola approach (D) thyroid surgery.

As shown in Figure 3, after the use of MHI during surgery, the lymph nodes were stained blue. MHI exhibited good staining effects in both conventional and endoscopic surgeries, with overall staining rates of 90.5 ± 8.83% and 92.03 ± 9.36%, respectively. In this study, all patients underwent unilateral or bilateral CND procedures. In statistical analysis, to facilitate the assessment of MHI's lymph node detection efficacy, unilateral CND was treated as a single observation, whereas bilateral CND was regarded as two separate observations, with each side of CND counted individually. Consequently, 396 observations were performed in the conventional surgery cohort (184 with MHI and 212 in the control group), and 187 observations were performed in the endoscopic surgery cohort (87 with MHI and 100 in the control group). The characteristics of harvested lymph nodes are summarized in Table 2. In the conventional surgery cohort, MHI increased the LNY from 4.59 to 5.89, but it did not significantly change the yield of metastatic lymph nodes. MHI mainly increased the yield of small (< 5mm) lymph nodes (2.75± 2.07 vs. 3.94± 2.66, *p* < 0.001) and did not significantly affect the yield of larger lymph nodes. In the endoscopic surgery cohort, MHI increased the LNY from 4.15 to 5.29 and mainly increased the yield of small (< 5mm) lymph nodes (2.17± 2.26 vs. 3.40± 2.27, *p* < 0.001), while the yield of larger lymph nodes remained unchanged. It can be seen that using MHI during surgery enhances the LNY, particularly for small non-metastatic lymph nodes, but does not enhance the detection rate of metastatic lymph nodes.

### MHI improves the protective effect on the inferior parathyroid glands

**Figure 4:**
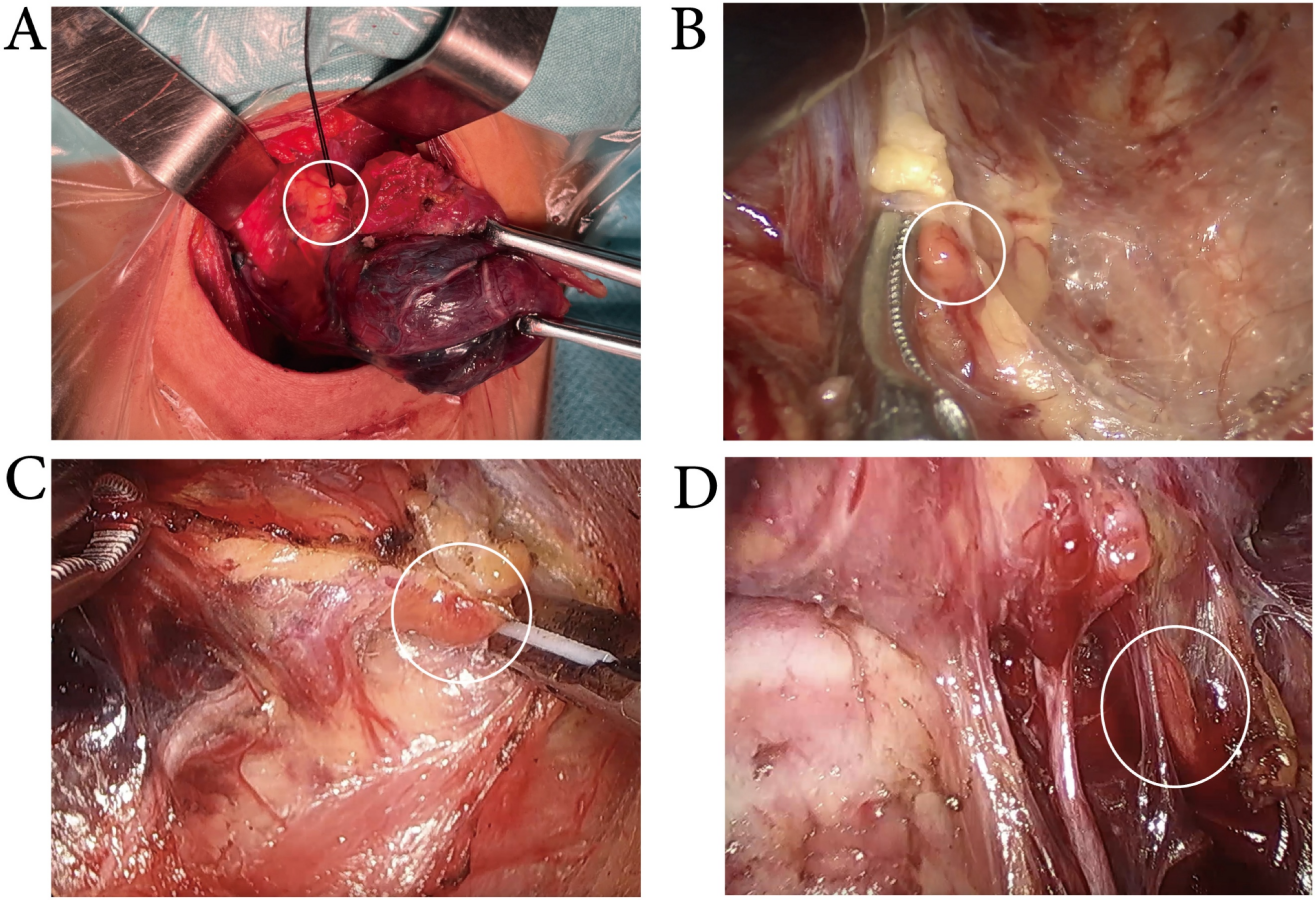
** Exposure of the superior PGs.** MHI did not improve the identification of the superior parathyroid glands, whether in conventional(A), transoral approach (B), transxillary approach (C), or bilateral areola approach (D) thyroid surgery.

**Figure 5:**
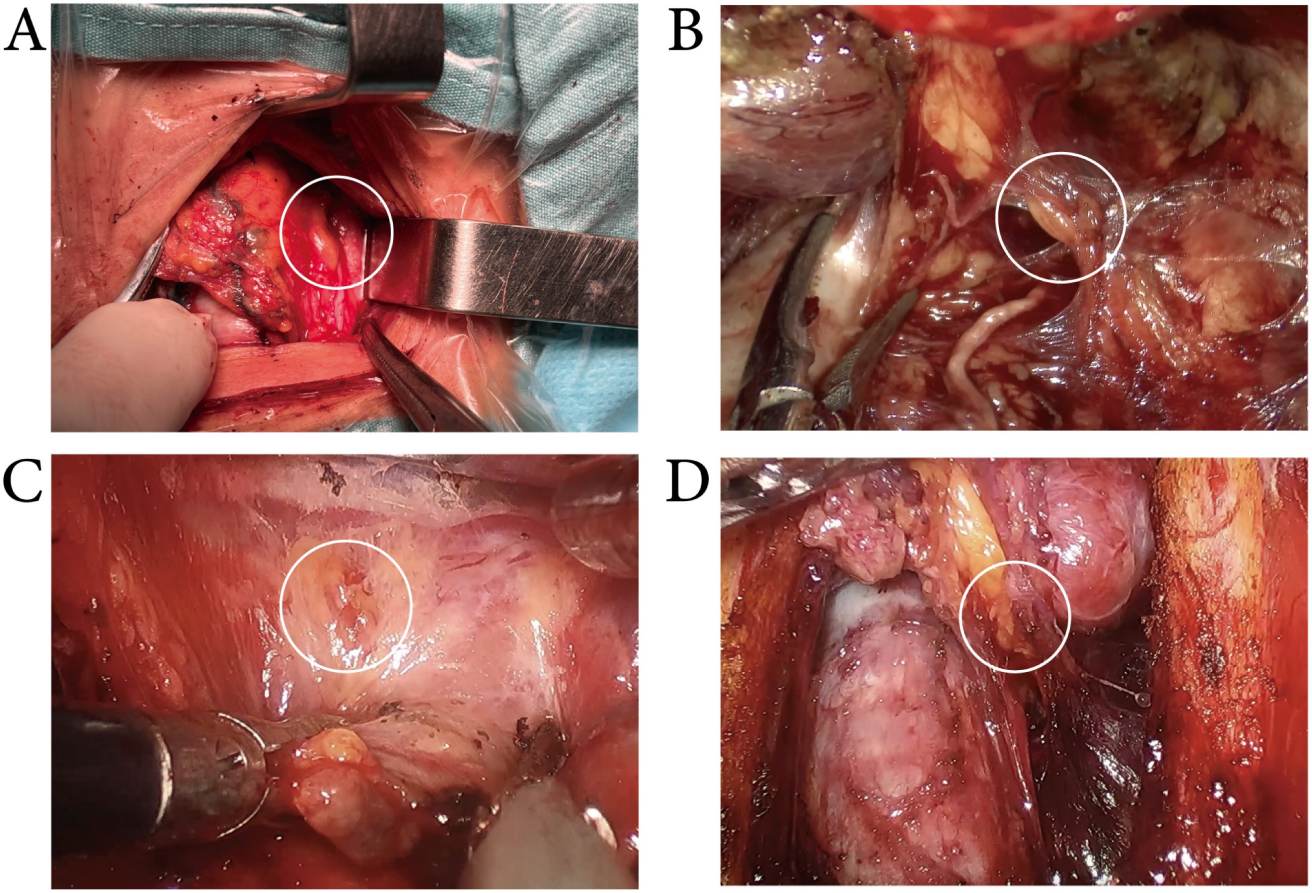
** Exposure of the inferior PGs.** While the lymph nodes were stained blue, the intraoperative identification and *in situ* preservation of inferior parathyroid glands were more easily achieved in conventional (A), transoral approach (B), transxillary approach (C), and bilateral areola approach(D) thyroid surgery.

For the data analysis of PGs, unilateral lobectomy combined with unilateral CND was defined as one observation of superior and inferior PG identification. Intraoperative PG identification was defined as either *in situ* preservation of the glands or autotransplantation of accidentally excised glands into the sternocleidomastoid muscle. Consequently, a total of 396 PG identification observations were recorded in the conventional surgery cohort, including 184 cases with MHI and 212 cases in the control group. Similarly, 187 PG identification observations were documented in the endoscopic thyroid surgery cohort, with 87 in the MHI group and 100 in the control group. The results related to PG identification are presented in Table 3. In the conventional surgery cohort, there were no significant differences in the identification, *in situ* preservation, or autotransplantation rates of superior PGs between the MHI and control groups. Similarly, in the endoscopic surgery cohort, there were no differences between the two groups (Figure 4). In the conventional surgery cohort, 60.87% inferior PGs were identified and 54.35% were preserved *in situ* in the MHI group. In the control group, 42.45% inferior PGs were identified and 37.74% were preserved *in situ*. In the endoscopic surgery cohort, MHI increased the identification rate from 41.00% to 60.92% and the preservation-in-situ rate from 32.00% to 47.12% (Figure 5). As presented in Table 1, preoperative, 1-day postoperative, and 30-day postoperative serum PTH levels were similar between the MHI and control groups in patients who underwent conventional or endoscopic surgery. In the conventional surgery cohort, 26 cases underwent transient hypoparathyroidism in the MHI group and 31 cases in the control group. In the endoscopic surgery cohort, 14 cases underwent transient hypoparathyroidism in the MHI group and 18 cases in the control group. No cases fulfilling criteria for permanent hypoparathyroidism during the follow-up period. The results show that MHI did not improve the identification of the superior PGs, which are relatively fixed in position. However, for the inferior PGs, whose position is more variable, MHI enhanced both the intraoperative identification and *in situ* preservation rates. Despite these improvements, MHI did not reduce the incidence of hypoparathyroidism.

## Discussion

Surgery is the primary treatment option for PTC 18. Dyes such as CNs, ICG, and MB have been used to improve thyroid surgery outcomes. CNs enter the lymphatic vessels, resulting in black staining of the thyroid gland and draining lymph nodes 19. However, according to Yu, accidental exudation of CNs during the operation can pollute the surgical field, making the surgical procedure quite challenging 20. In addition, CNs remain in the body for a long time, and the black dye in the field makes reoperation more challenging. Wu described cases in which nanocarbon suspension caused carbon deposition in the trachea, leading to progressive dyspnea 21. Once injected, ICG binds rapidly to plasma proteins and fluoresces under near-infrared light, allowing detection by the imaging system 22. Thus, ICG enables the visualization of the PGs blood vessels. ICG requires additional fluorescence imaging systems, which are often unavailable in most hospitals, and the dim light may impede operative visualization and increase procedural complexity. It is also prohibited in patients with iodine allergy and renal dysfunction 23. In the present study, no MHI-associated complications were documented, a finding that aligns well with the conclusions of prior research 12,24,25. The lymph nodes in the central neck compartment were stained blue without disturbing the surgical field. The use of MHI did not introduce additional challenges to the procedure, either in conventional or endoscopic surgery.

It' reported that higher LNY in CNDs correlates with lower rates of PTC recurrence in the central neck 26. As mentioned previously, multiple tracers were used to harvest more lymph nodes. Studies have shown that CNs can increase the LNY and the number of detected metastatic lymph nodes, but they do not alter the proportion of metastatic nodes within the total harvested lymph nodes 27,28. We found that MHI can enhance LNY. However, MHI primarily increased the identification of non-metastatic lymph nodes smaller than 5 mm, without improving the detection of metastatic lymph nodes. This suggests that MHI may primarily enhance the identification of lymph nodes in postoperative specimens through staining, rather than facilitating the detection of metastatic nodes. In this study, all enrolled patients underwent prophylactic or therapeutic CND, with relatively few cases of locally advanced thyroid cancer. Whether MHI can improve the detection of metastatic lymph nodes in advanced thyroid cancer requires further exploration.

The clinical significance of MHI in improving the detection rate of small non-metastatic lymph nodes (< 5 mm) appears to lie primarily in assisting pathologists in identifying minute lymph nodes in surgical specimens, thereby reducing pathological assessment bias caused by lymph node omission. This is particularly valuable for evaluating the completeness of CND. According to the current risk stratification system for thyroid cancer, although the detection of small non-metastatic lymph nodes does not alter the tumor stage, it can enhance the accuracy of N-stage assessment and reduce the proportion of “pNx” (regional lymph node status unknown), which is especially relevant for prognostic evaluation in patients with low-risk PTC. Additionally, adequate dissection of small lymph nodes may mitigate the risk of potential occult metastasis. Although this study did not show an improvement in the detection rate of metastatic lymph nodes, comprehensive central neck lymph node assessment remains an important basis for formulating postoperative management plans in patients with small tumors and no obvious evidence of metastasis.

Hypoparathyroidism is another common complication of thyroid surgery. Shoback DM *et al.* reported that following total thyroidectomy, 20%- 30% of patients experience transient hypoparathyroidism, while 1%-7% develop permanent hypoparathyroidism 29. This is mainly due to the variable anatomical location of the parathyroid glands, especially the lower two, which increases the risk of accidental removal during thyroid surgery 30. A review summarizes the intraoperative methods for identifying PGs 31. Near-infrared autofluorescence enables real-time identification and localization of parathyroid glands during surgery, reducing accidental removal and autotransplantation of PGs. ICG fluorescence imaging allows visualization of parathyroid gland blood vessels, assessment of gland viability, guidance for autotransplantation, and prediction of postoperative hypoparathyroidism. CNs reduce the risk of accidental removal of a PG by 30%- 34% and lower the incidence of transient hypoparathyroidism by 46% 27,28. In this study, the recognition rate of the superior PGs was high, and MHI did not enhance its recognition efficacy. This may be related to the relatively fixed position of the superior PGs, the less surrounding adipose tissue, and the ease of exposure during surgery. MHI significantly promoted the recognition of the inferior PGs. Compared with the control group, the identification rate in the MHI group increased by 18.42%- 19.92%, and more inferior PGs are preserved *in situ*. This may be attributed to the “negative contrast” effect formed by the blue staining of MHI, which facilitates the differentiation of parathyroid glands from lymph nodes and the surrounding adipose and connective tissues. This effect helps surgeons to more precisely delineate the dissection boundary intraoperatively, thereby preventing excessive injury to adjacent normal tissues—particularly the parathyroid glands and their supplying blood vessels. Ultimately, the incidence of transient and permanent hypoparathyroidism did not differ significantly between the two cohorts. This observation is likely attributable to our institution's consistent focus on the exposure and functional preservation of superior PGs—a practice honed by our extensive experience in high-volume thyroid surgery. Surgeons at our center have achieved proficiency in identifying the anatomical structure of the parathyroid glands and mastering parathyroid function preservation techniques, resulting in a low baseline risk of parathyroid injury. This established advantage may have blunted the incremental clinical benefits that might otherwise be conferred by MHI. Additionally, although the sample size of this study is relatively large, the incidence of transient hypoparathyroidism is low, which may lead to insufficient statistical power to detect subtle differences. Future studies with an expanded sample size will be conducted to further validate these findings.

## Conclusion

This study retrospectively compared the clinical application of MHI in both conventional and endoscopic thyroid surgeries. It first established the clinical safety of a novel lymph node tracer for thyroid surgery, MHI. Subsequently, the study evaluated the role of MHI in enhancing the LNY in the central neck compartment during thyroid surgery. The results showed that MHI could improve the LNY, but this was limited to non-metastatic lymph nodes smaller than 5 mm, with no significant benefit in detecting metastatic lymph nodes. Finally, the study confirmed that MHI significantly improved the intraoperative identification and *in situ* preservation rates of inferior PGs during thyroid surgery, although its protective effect on parathyroid function during the recovery phase was limited. The study did not include patients with locally advanced thyroid cancer, and further research is needed to explore the protective role of MHI in this group of patients.

## Figures and Tables

**Table 1:** Baseline characteristics in MHI group versus control group

	Conventional Sugery			Endoscopic Sugery		
	MHI group (n = 120)	Control group (n = 138)	*p* value	MHI group (n = 65)	Control group (n = 74)	*p* value
Age (years)	43.82± 11.67	43.80± 9.66	0.99	35.31± 9.56	35.92± 7.99	0.68
Gender (n)			0.06			0.62
Male	27 (22.50%)	44 (31.88%)		7 (10.77%)	10 (13.51%)	
Female	93 (77.50%)	94 (68.12%)		58 (89.23%)	64 (86.49%)	
Extent of Surgery (n)			0.94			0.34
HT+ CND	49 (40.83%)	57 (41.30%)		37 (56.92%)	48 (64.86%)	
TT+ CND	71 (59.17%)	81 (58.70%)		28 (43.08%)	26 (35.14%)	
Scope of CND (n)			0.96			0.87
Unilateral	56 (46.67%)	64 (46.38%)		43 (66.15%)	48 (64.86%)	
Bilateral	64 (53.33%)	74 (53.26%)		22 (33.85%)	26 (35.14%)	
Type of CND (n)			0.61			0.79
Therapeutic CND	32 (26.67%)	33 (23.91%)		12 (18.46%)	15 (20.27%)	
Prophylactic CND	88 (73.33%)	105 (76.09%)		53 (81.54%)	59 (79.73%)	
Operation Duration (min)	86.12± 20.57	87.72± 16.33	0.49	129.42± 37.63	123.07± 38.40	0.98
Surgical approaches (n)	-	-	-			
Transoral				32 (49.23%)	39 (52.70%)	0.68
Transaxillary				22 (33.85%)	24 (32.43%)	0.86
Bilateral areola				11 (16.92%)	11 (14.86%)	0.74
Tumor size (mm)	12.27± 9.42	11.01± 5.45	0.19	9.93± 7.76	9.72± 4.66	0.84
Number of tumor (n)	1.55± 0.99	1.45± 0.77	0.39	1.38± 0.68	1.22± 0.53	0.10
Multifocal tumor (n)			0.90			0.10
Yes	40 (33.33%)	45 (32.61%)		19 (29.23%)	13 (17.57%)	
No	80 (66.67%)	93 (67.39%)		46 (70.77%)	61 (82.43%)	
Tumor location (n)			0.96			0.10
Unilateral	90 (75.00%)	103 (74.64%)		46 (70.77%)	61 (82.43%)	
Bilateral	30 (25.00%)	35 (25.36%)		19 (29.23%)	13 (17.57%)	
Extrathyroidextension (n)			0.28			0.61
Yes	33 (27.50%)	30 (21.74%)		11 (16.92%)	15 (20.27%)	
No	87 (72.50%)	108 (78.26%)		54 (83.08%)	59 (79.73%)	
Metastatic lymph node^a^ (n)			0.99			0.18
Yes	65 (54.17%)	75 (54.35%)		34 (52.31%)	47 (63.51%)	
No	55 (45.83%)	63 (45.65%)		31 (47.69%)	27 (36.49%)	
Preoperative PTH (pg/ml)	43.64± 13.95	44.30± 17.50	0.72	43.32± 14.02	39.22± 12.41	0.07
Preoperative Calcium (mmol/L)	2.33± 0.24	2.32± 0.23	0.84	2.30± 0.31	2.33± 0.10	0.38
PTH on POD 1 (pg/ml)	20.76± 14.95	23.68± 14.36	0.11	24.87± 15.85	27.30± 10.74	0.29
Calcium on POD 1 (mmol/L)	2.22± 0.13	2.23± 0.14	0.32	2.21± 0.11	2.22± 0.13	0.48
PTH on POD 30 (pg/ml)	37.13± 14.21	41.42± 33.51	0.19	36.23± 13.73	35.20± 11.46	0.63
Calcium on POD 30 (mmol/L)	2.30± 0.13	2.29± 0.10	0.41	2.32± 0.11	2.29± 0.07	0.07
Hypoparathyroidism on POD 1 (n)	26 (21.67%)	31 (22.46%)	0.88	14 (21.54%)	18 (24.32%)	0.70
Hypoparathyroidism on POD 30 (n)	1 (0.83%)	1 (0.72%)	1.00	1 (1.54%)	3 (4.05%)	0.62
Hypoparathyroidism on POM 6 (n)	0	0	-	0	0	-
Serious complications (n)	5 (4.16%)	6 (4.35%)	0.94	2 (3.08%)	6 (8.11%)	0.28
Hematoncus	1	1		0	1	
Hoarseness	3^b^	5^b^		2	4	
Others	1^c^	0		0	1^d^	

TT, total thyroidectomy; HT, hemithyroidectomy; CND, central neck dissection; POD, postoperative day; POM, postoperative month; ^a^Metastatic lymph node (Yes/No) refers exclusively to central compartment lymph node metastasis. ^b^ In two cases, extrathyroidextension of the recurrent laryngeal nerve caused permanent hoarseness. ^c^ Infection of incisional wound. ^d^ Subcutaneous emphysema.

**Table 2:** Lymph node yield per unilateral central neck in MHI group versus control group

	Conventional Sugery			Endoscopic Sugery		
	MHI group (n^#^ = 184)	Control group (n^#^ = 212)	*p* value	MHI group (n^#^ = 87)	Control group (n^#^ = 100)	*p* value
Lymph node yield (n)	5.89± 3.51	4.59± 2.74	< 0.001*	5.29± 2.62	4.15± 2.87	< 0.001*
Metastic lymph nodes (n)	1.71± 1.99	1.46± 1.58	0.18	1.67± 2.07	1.50± 1.93	0.57
Diameter of lymph nodes (n)						
< 5mm	3.94± 2.66	2.75± 2.07	< 0.001*	3.40± 2.27	2.17± 2.26	< 0.001*
5- 10mm	1.78± 2.31	1.64± 1.92	0.52	1.31± 2.11	1.65± 1.98	0.26
> 10mm	0.17± 0.48	0.16± 0.46	0.78	0.18± 0.46	0.23± 0.76	0.62

^#^ n represents the number of observations, with unilateral central neck dissection counted as one observation and bilateral dissection as two; * p < 0.05

**Table 3:** Identification of parathyroid glands per unilateral central neck in MHI group versus control group

	Conventional Sugery			Endoscopic Sugery		
	MHI group (n^#^ = 184)	Control group (n^#^ = 212)	*p* value	MHI group (n^#^ = 87)	Control group (n^#^ = 100)	*p* value
Superior PGs (n)						
Identified	160 (86.95%)	183 (86.32%)	0.85	78 (89.66%)	85 (85.00%)	0.34
Preserved *in situ*	148 (82.43%)	169 (79.72%)	0.86	65 (74.71%)	72 (72.00%)	0.68
Autotransplanted	12 (6.52%)	14 (6.60%)	0.97	13 (14.94%)	13 (13.00%)	0.70
Inferior PGs (n)						
Identified	112 (60.87%)	90 (42.45%)	< 0.001*	53 (60.92%)	41 (41.00%)	0.01*
Preserved *in situ*	100 (54.35%)	80 (37.74%)	< 0.001*	41 (47.12%)	32 (32.00%)	0.03*
Autotransplanted	12 (6.52%)	10 (4.71%)	0.43	12 (13.79%)	9 (9.00%)	0.30

PGs, parathyroid glands; ^#^ n represents the number of observations for parathyroid gland identification; unilateral lobectomy combined with unilateral central neck dissection was defined as one observation. * *p*< 0.05

## Abbreviations

CN: carbon nanoparticle
CND: central neck dissection
ETBAA: endoscopic thyroidectomy via bilateral areola approach
ICG: indocyanine green
LNY: Lymph node yield
MB: methylene blue
MHI: Mitoxantrone Hydrochloride Injection
PG: parathyroid gland
PTC: papillary thyroid cancer
PTH: parathyroid hormone
TAT: transaxillary approach thyroidectomy
TOETVA: transoral endoscopic thyroidectomy via the vestibular approach
